# l-Lactic acid production from glucose and xylose with engineered strains of *Saccharomyces cerevisiae*: aeration and carbon source influence yields and productivities

**DOI:** 10.1186/s12934-018-0905-z

**Published:** 2018-04-11

**Authors:** Vera Novy, Bernd Brunner, Bernd Nidetzky

**Affiliations:** 1 Institute of Biotechnology and Biochemical Engineering, NAWI Graz, Graz University of Technology, Petersgasse 12/I, 8010 Graz, Austria; 2 Austrian Centre of Industrial Biotechnology, Graz, Austria

**Keywords:** l-Lactic acid production, Xylose fermentation, *Saccharomyces cerevisiae*, Lactate dehydrogenase, Pyruvate decarboxylase, Pyruvate branching point

## Abstract

**Background:**

*Saccharomyces cerevisiae*, engineered for l-lactic acid production from glucose and xylose, is a promising production host for lignocellulose-to-lactic acid processes. However, the two principal engineering strategies—pyruvate-to-lactic acid conversion with and without disruption of the competing pyruvate-to-ethanol pathway—have not yet resulted in strains that combine high lactic acid yields (*Y*_LA_) and productivities (Q_LA_) on both sugar substrates. Limitations seemingly arise from a dependency on the carbon source and the aeration conditions, but the underlying effects are poorly understood. We have recently presented two xylose-to-lactic acid converting strains, IBB14LA1 and IBB14LA1_5, which have the l-lactic acid dehydrogenase from *Plasmodium falciparum* (*pf*LDH) integrated at the *pdc1* (pyruvate decarboxylase) locus. IBB14LA1_5 additionally has its *pdc5* gene knocked out. In this study, the influence of carbon source and oxygen on *Y*_LA_ and Q_LA_ in IBB14LA1 and IBB14LA1_5 was investigated.

**Results:**

In anaerobic fermentation IBB14LA1 showed a higher *Y*_LA_ on xylose (0.27 g g_Xyl_^−1^) than on glucose (0.18 g g_Glc_^−1^). The ethanol yields (*Y*_EtOH_, 0.15 g g_Xyl_^−1^ and 0.32 g g_Glc_^−1^) followed an opposite trend. In IBB14LA1_5, the effect of the carbon source on *Y*_LA_ was less pronounced (~ 0.80 g g_Xyl_^−1^, and 0.67 g g_Glc_^−1^). Supply of oxygen accelerated glucose conversions significantly in IBB14LA1 (Q_LA_ from 0.38 to 0.81 g L^−1^ h^−1^) and IBB14LA1_5 (Q_LA_ from 0.05 to 1.77 g L^−1^ h^−1^) at constant *Y*_LA_ (IBB14LA1 ~ 0.18 g g_Glc_^−1^; IBB14LA1_5 ~ 0.68 g g_Glc_^−1^). In aerobic xylose conversions, however, lactic acid production ceased completely in IBB14LA1 and decreased drastically in IBB14LA1_5 (*Y*_LA_ aerobic ≤ 0.25 g g_Xyl_^−1^ and anaerobic ~ 0.80 g g_Xyl_^−1^) at similar Q_LA_ (~ 0.04 g L^−1^ h^−1^). Switching from aerobic to microaerophilic conditions (pO_2_ ~ 2%) prevented lactic acid metabolization, observed for fully aerobic conditions, and increased Q_LA_ and *Y*_LA_ up to 0.11 g L^−1^ h^−1^ and 0.38 g g_Xyl_^−1^, respectively. The *pf*LDH and PDC activities in IBB14LA1 were measured and shown to change drastically dependent on carbon source and oxygen.

**Conclusion:**

Evidence from conversion time courses together with results of activity measurements for *pf*LDH and PDC show that in IBB14LA1 the distribution of fluxes at the pyruvate branching point is carbon source and oxygen dependent. Comparison of the performance of strain IBB14LA1 and IBB14LA1_5 in conversions under different aeration conditions (aerobic, anaerobic, and microaerophilic) further suggest that xylose, unlike glucose, does not repress the respiratory response in both strains. This study proposes new genetic engineering targets for rendering genetically engineering *S. cerevisiae* better suited for lactic acid biorefineries.

**Electronic supplementary material:**

The online version of this article (10.1186/s12934-018-0905-z) contains supplementary material, which is available to authorized users.

## Background

l-Lactic acid is an industrially important bulk chemical and has received increasing attention as a precursor for the production of the bioplastic polymer poly-lactic acid [1–3]. To meet the growing demand, sustainably and environmentally compatible lactic acid production from renewable resources, ideally from lignocellulosic waste streams, must be facilitated [1]. This necessitates a fermentation organism, which is robust, pH stable, and capable of efficient conversion of the two main lignocellulosic sugars glucose and xylose [1, 2, 4]. Because currently applied lactic acid producing organisms (e.g., lactic acid bacteria) do not meet these requirements [1–3], research has focused on engineering novel strains [1–5]. *Saccharomyces cerevisiae* thereby represents the most prominent host organism, due to its suitable pH tolerance, the high operational stability, and the availability of a genetic engineering toolbox [1–3]. Extensive research within the field of lignocellulose-to-bioethanol processes has further generated *S. cerevisiae* strains capable of efficiently metabolizing xylose [6, 7], even in the harsh environment presented by the lignocellulosic substrates [8].

**Fig. 1:**
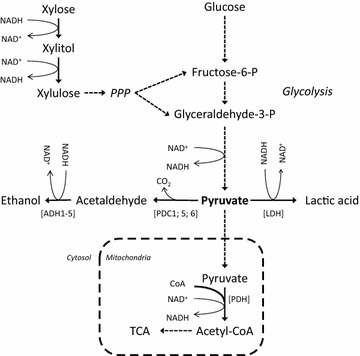
Schematic representation of the metabolic pathways at the pyruvate branching point in *S. cerevisiae* engineered for conversion of glucose and xylose to l-lactic acid. Pathways are indicated with dashed arrows. Single enzyme reactions are represented in full arrows with the participating enzymes specified in brackets. *ADH1-5* alcohol dehydrogenase 1 to 5, *LDH* lactate dehydrogenase, *PDC1; 5; 6* pyruvate decarboxylase 1, 5 and 6, *PPP* pentose phosphate pathway

The lack of lactic acid pathways in natural strains of *S. cerevisiae* requires the introduction of the gene encoding for l-lactic acid dehydrogenase (LDH) [3, 9–12]. LDH catalyzes the reduction of pyruvate by NADH to yield l-lactic acid (Fig. 1). Pioneering studies showed that *ldh*-harboring strains of *S. cerevisiae* converted glucose to lactic acid [13, 14], but also revealed the main challenge with this approach, which is the formation of ethanol at the expense of lactic acid yields and productivities [3, 9–12, 15]. At the pyruvate branching point, the LDH is in direct competition for pyruvate with the pyruvate decarboxylases (PDC, Fig. 1). It also competes for NADH with the alcohol dehydrogenases (ADH, Fig. 1). To channel the metabolic flux away from ethanol towards lactic acid production, further engineering strategies were applied. These included (a) the alleviation or disruption of the ethanol pathway by gene deletion of *pdc1*, *pdc5*, *pdc6*, *adh1*, or a combination thereof [9, 10, 12, 16]; (b) the optimization of *ldh* gene expression [11, 17, 18], (c) the application of LDHs with high catalytic activities [19, 20]; (d) the perturbation of the intracellular redox balance to increase the availability of NADH for the LDH-catalyzed reaction [18]; (e) the reduction of by-product formation by disruption of the glycerol pathway [21], and (f) the reduction of ATP consumption by replacing the native with an ATP-independent pathway for acetyl-CoA production from acetaldehyde [15, 22]. Recent studies by us [20] and others [23] further showed that xylose-to-lactic acid conversion in *S. cerevisiae* is possible, representing an important step to advance lignocellulose-to-lactic acid processes.

Despite these achievements, there are still challenges inherent to the yeast’s metabolism, which restrict the yields, the productivities, or both. Thus, homolactate fermentation on glucose was so far only achieved with PDC-deficient strains of *S. cerevisiae* [10, 12, 16]. These strains, however, showed severe distortion of biomass growth and cell viability [20, 24–26]. Further, under anaerobic conditions, the preferred set-up for larger scale applications, only low productivities were obtained and these typically declined rapidly over fermentation time [2, 10, 20, 25]. Previous studies suggested that lactic acid production in recombinant *S. cerevisiae* does not yield net ATP, likely because the generated ATP is consumed largely to manage export of lactic acid out of the cell [25, 27]. Under absence of oxygen, the cells are thus unable to meet the ATP requirements for maintenance.

Extending the substrate scope towards xylose brings additional challenges. Turner et al. have recently presented the *S. cerevisiae* strain SR8L, which comprises the two-step xylose pathway based on xylose reductase (XR) and xylitol dehydrogenase (XDH) together with an intact pyruvate-to-ethanol pathway [23]. When xylose was the substrate, SR8L showed progress towards homolactate conversion with *Y*_LA_ of up to 0.7 g g_Xyl_^−1^. However, under anaerobic conditions or when glucose was the substrate, mixed lactic acid and ethanol formation was observed [23].

A dependency of both *Y*_LA_ and lactic acid productivity (Q_LA_) on oxygen supply by aeration was also noted in a recent study from the current authors, in which two strains of *S. cerevisiae*, IBB14LA1 and IBB14LA1_5, producing lactic acid from xylose were presented [20]. Both these strains are descendants of strain IBB10B05, which harbors a XR/XDH pathway engineered for redox-neutral assimilation of xylose and has furthermore undergone evolution for accelerated xylose-to-ethanol fermentation [28, 29]. IBB14LA1 was derived from strain IBB10B05 by introduction of the *pf*LDH at the *pdc1* locus [20]. Due to high ethanol formation, IBB14LA1 was previously not analyzed in more detail. Instead the *pdc5* gene was deleted in IBB14LA1, resulting in IBB14LA1_5, which showed excellent *Y*_LA_ (> 0.7 g g^−1^) in anaerobic fermentations, independent of the carbon source used [20]. Switching from anaerobic to aerobic conditions boosted the lactic acid productivity on glucose substantially (Q_LA_ from 0.07 to 1.8 g L^−1^ h^−1^), but caused the xylose-to-lactic acid conversion to cease almost completely [20]. These effects were not well understood. The aim of the current study therefore was to further investigate the influence of carbon source and oxygen on *Y*_LA_ and Q_LA_ in xylose-fermenting *S. cerevisiae*, comparing strains with intact (IBB14LA1) and disrupted (IBB14LA1_5) pyruvate-to-ethanol pathway.

## Methods

### Strains and media

The *S. cerevisiae* strains IBB14LA1 (*pdc1*::*pfldh*) and IBB14LA1_5 (*pdc1*::*pfldh*, *Δpdc5*) were utilized. They were derived from the genetically and evolutionary engineered strain IBB10B05, which is able to ferment xylose [28]. A detailed description of strain construction is given elsewhere [20]. In brief, the gene encoding for the LDH from *Plasmodium falciparum* was integrated at the *pdc1* locus under control of the *pdc1* promotor, replacing the native *pdc1* gene (IBB14LA1). Strain IBB14LA1_5 was derived from IBB14LA1 by additional deletion of the *pdc5* gene.

Seed and starter cultures were prepared in YP media (10 g L^−1^ yeast extract, 20 g L^−1^ peptone from casein), containing either 20 g L^−1^ glucose (YPD) or 10 g L^−1^ ethanol (YPE). Conversion experiments were conducted in complex media (10 g L^−1^ yeast extract) supplemented with either glucose (YG) or xylose (YX) at 50 g L^−1^ each. CaCO_3_ (11 g L^−1^) was added to all conversion experiments, anaerobic and aerobic. The CaCO_3_ was shown to effectively stabilize the pH under these conditions [20]. All chemicals were from Carl Roth + Co KG (Karlsruhe, Germany).

### Anaerobic, aerobic and microaerophilic cultivations

Anaerobic cultivations were performed in glass bottles tightly sealed with rubber septa (90 mL working volume). Incubation was at 30 °C and 190 rpm (CERTOMAT BS-1, Sartorius AG, Göttingen, Germany). Aerobic conversions were conducted in 300 mL baffled shaken flasks filled with 50 mL media, loosely closed with cotton foam to provide sufficient oxygen supply. Incubation was at 30 °C and ~ 110 rpm. The starting OD_600_ of anaerobic and aerobic cultivations was 5. For both set-ups, cell propagation was performed in seed cultures followed by starter cultures, which were performed in baffled shaken flasks containing YPD (IBB14LA1) or YPE (IBB14LA1_5) media. A detailed description of the experimental procedure can be found elsewhere [20].

Microaerophilic conversions were conducted in 2 L Labfors III bioreactors (Infors AG, Bottmingen, Switzerland), equipped with off-gas ethanol and CO_2_ analyzers (Innova 1313, LumaSense Technologies A/S, Frankfurt am Main, Germany). The conversions were run in two phases. An aerobic biomass production phase with YPE media (1 L working volume) was followed by a microaerophilic conversion phase with YX media (2 L). The conditions were 30 °C and pH 6.8, continuously adjusted with 5 M NaOH. The dissolved oxygen concentration (pO_2_) was 50% for the biomass production phase, and 2% for the conversion phase. The pO_2_ was controlled online with an agitation (100–1000 rpm) and aeration (0.16–2 L min^−1^ pressurized air) cascade. Seed cultures were prepared in shaken flasks (300 mL), filled with 50 mL YPE medium. Cells were transferred to the bioreactor to a starting OD_600_ of 0.5. When an OD_600_ of ~ 10 was reached, the conversion phase was initiated by adding 1 L of YX media and reducing the pO_2_ to 2%.

### Sampling, analysis of metabolites and data evaluation

Samples were taken regularly from all experiments. Immediate sample-work up included the measurement of the OD_600_ and pH in the cell suspension. One mL of the samples was further centrifuged (15,700*g*, 4 °C, 10 min, 5415 R; Eppendorf, Hamburg, Germany) and the supernatant stored at − 20 °C for HPLC analysis, which was used to quantify glucose, xylose and metabolites (LA, ethanol, xylitol, glycerol, acetate and pyruvate). Reported yield coefficients were based on mass. In aerobic and anaerobic cultivations without off-gas analysis, CO_2_ formation was included in carbon recoveries by assuming that 1 mol CO_2_ was formed per mol acetate and ethanol produced. For biomass yields a value of 26.4 g Cmol^−1^ was applied [30]. Q_Xyl_ and Q_LA_ are given in g L^−1^ h^−1^. A detailed description of the HPLC setup and data evaluation can be found elsewhere [20].

### Analysis of enzyme activities

*pf*LDH and PDC activities were measured in cell extract of strain IBB14LA1 cultivated under anaerobic and aerobic conditions on glucose and xylose. As control, PDC activities were additionally measured in the cell extracts of the parent strain IBB10B05. All measurements were done in quadruplicates, representing biological and technical replicates. Cells were harvested from culture suspensions (50 mL) at the indicated time points using centrifugation (4500*g*, 10 min, 4 °C, 5810 R Eppendorf). The cell pellet was resuspended in 50 mL of 0.2 M HCl to dissolve CaCO_3_. After centrifugation (4500*g*, 10 min, 4 °C), the cells were taken up in 50 mL 0.9% NaCl solution and divided into 10 mL aliquots, in which the cell densities (OD_600_) were determined. For *pf*LDH and PDC activity measurements, the cells were disrupted mechanically in sodium phosphate buffer (Na–Ac, 0.1 M, pH 7.5) and imidazole–HCl buffer (I–HCl, 240 mM, pH 6.5), respectively, each supplemented with protease inhibitor (“cOmplete”, Roche, Basel, Switzerland). Both activities were determined in the cell extract as the change of absorbance at 340 nm over time (25 °C, DU800 spectrophotometer, Beckam Coulter Inc., Brea, CA). The *pf*LDH activity was measured in Na–Ac buffer, supplemented with sodium pyruvate (1 mM) and NADH (0.4 mM). The *pf*LDH activity was also measured in the cell extract of IBB10B05, where no drop in NADH concentration was detected, thus excluding unspecific NADH consumption by other cell enzymes. For the PDC activity measurement, I–HCl buffer additionally containing thiamine pyrophosphate (0.2 mM), MgCl (5 mM), NADH (0.15 mM), sodium pyruvate (50 mM), and ADH (~ 3.5 U/mL, Sigma-Aldrich, St. Louis, MO) was used. Analysis by HPLC of the reaction mixture incubated in the presence of IBB14LA1 cell extract showed that lactic acid was produced under these conditions. This result indicated that the PDC assay involved a problem from the competing reaction of the *pf*LDH which utilizes pyruvate and consumes NADH. To nonetheless assess the PDC activity in strain IBB14LA1, the reaction mixtures were put on ice immediately after spectrophotometric measurements and then stored at − 20 °C for the determination of the lactic acid concentration by HPLC. This was then used to calculate the amount of NADH consumed by the *pf*LDH reaction, which was subtracted from the apparent PDC activity (i.e., the total NADH consumption in the assay). PDC activities that were thus determined are denoted PDC*corr*. PDC activity measured in IBB10B05, which does not harbor the *pfldh* gene and so lacks the competing reaction from LDH, is denoted PDC*dir*. The concentration of the crude cell protein was measured with the Roti-Quant assay (Carl Roth Gmbh & Co. Kg, Karlsruhe, Germany) using BSA standard. All activities are given in Units (U) mg_crude cell protein_^−1^, with 1 U being defined as the conversion of 1 μmol of NADH per minute.

## Results and discussion

### Strain IBB14LA1 harboring an intact pyruvate-to-ethanol pathway shows *Y*_LA_ dependent on the carbon source

**Fig. 2:**
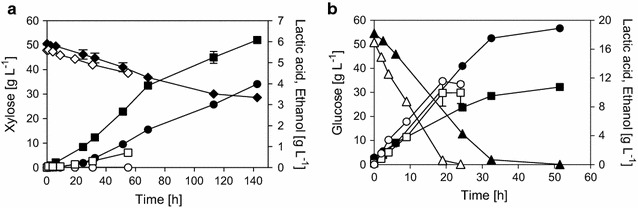
Time courses of xylose (**a**) and glucose (**b**) conversions using IBB14LA1. Depicted are the xylose (**a**, diamonds) and glucose (**b**, triangles) consumption and the lactic acid (**a**, **b**, squares) and ethanol (**a**, **b**, circles) production in anaerobic (**a**, **b**, filled symbols) and aerobic (**a**, **b**, empty symbols) conversion experiments. Data and error bars represent mean values and the spread of biological duplicates. Data for anaerobic conversions are taken from [20]

**Fig. 3:**
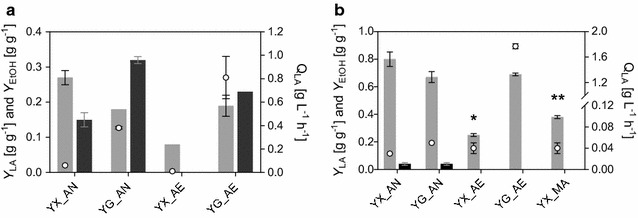
The influence of carbon source and aeration conditions on *Y*_LA_ (black bars), *Y*_EtOH_ (grey bars) and Q_LA_ (empty circles) in IBB14LA1 (**a**) and IBB14LA1_5 (**b**). Experiments were conducted in glucose-(YG) or xylose-(YX) based media under aerobic (_AE), anaerobic (_AN) or microaerophilic (_MA) conditions, as indicated. Data for YX_AE (marked with asterisk) were determined for 41 h of conversion time, before lactic acid uptake started (Fig. 5a and Table 1). Data for YX_MA (marked with double asterisk) represent parameters for phase II of the bioreactor conversion (Fig. 6 and Table 1). Data and error bars represent the mean values and the spread of biological duplicates. Data for YG_AE, YG_AN and YX_AN were taken from [20]

IBB14LA1 was firstly characterized in conversions of glucose- and xylose-based media (YG and YX) under anaerobic conditions. The respective time courses of substrate consumption and product formation are shown in Fig. 2 and a summary of physiological parameters calculated from the time course data can be found in Additional file 1: Table S1. The parameters *Y*_LA_, *Y*_EtOH_, and Q_LA_ are compared in Fig. 3a. The results reveal a clear change in *Y*_LA_, and in *Y*_EtOH_ in opposite direction, in dependence of the carbon source. Thus, on xylose, lactic acid (*Y*_LA_ 0.27 g g_Xyl_^−1^) was the main product and ethanol (*Y*_EtOH_ 0.15 g g_Xyl_^−1^) the main by-product (Fig. 2a). When glucose was the carbon source, however, IBB14LA1 produced more ethanol (*Y*_EtOH_ 0.32 g g_Glc_^−1^) than lactic acid (*Y*_LA_ 0.18 g g_Glc_^−1^, Fig. 2b). These findings imply that the distribution of fluxes at the pyruvate branching point in IBB14LA1 was governed by the carbon source.

The *S. cerevisiae* strain SR8L [23] showed a pattern of anaerobic conversion of xylose and glucose similar to that of IBB14LA1. To explain the effect of sugar substrate on *Y*_LA_ in strain SR8L, the authors suggested that slower xylose as compared to glucose utilization could have resulted in comparably lower pyruvate levels in the cell. Based on the smaller pyruvate *K*_M_ for the *ro*LDH (1.30 mM, [31]) as compared to the PDC (~ 2 mM, [32]), this would favor the LDH over the PDC reaction, so resulting in increased *Y*_LA_ and decreased *Y*_Ethanol_ [23].

However, IBB14LA1 harbors the *pf*LDH, which has a 61-fold higher catalytic efficiency (i.e. *k*_cat_/*K*_M_) for pyruvate than the *ro*LDH introduced into strain SR8L [20, 23, 31]. We have shown in a previous study that, assuming an intracellular pyruvate and NADH concentration of 0.6 and 0.3 mM, respectively [33, 34], the specific catalytic rate, *v*/[E] = *k*_cat_ × [S]/(*K*_M_ + [S]), of the *pf*LDH exceeds that of the PDC5 by one order of magnitude [20]. PDC5 is the principally expressed PDC in the *pdc1*-deficient IBB14LA1 [26, 35]. It therefore seems extremely unlikely that, assuming constant LDH/PDC protein ratios in the cell, the change in the intracellular pyruvate concentration could have affected the relative specific rates of the two enzymes to the extent observed (Fig. 3a). Since besides pyruvate the *pf*LDH also requires NADH for activity, the specific rate of lactic acid formation might also be limited by the coenzyme. A previous study has shown that enhancement of the NADH availability, caused by the perturbation of the intracellular NADH/NAD^+^ ratio, can improve lactic acid production from glucose by *S. cerevisiae* [18]. However, considering the kinetic parameter of *pf*LDH (*K*_M_-NADH 0.01 mM, *K*_M_-Pyruvate 0.03 mM, and *k*_cat_ 450 s^−1^ [36]), the specific catalytic rate should be rather insensitive, even to a 10-fold decrease in the NADH concentration from 0.3 mM (as described in [33]) to 0.03 mM.

**Fig. 4:**
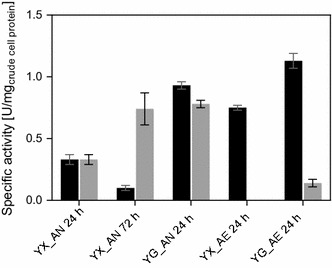
The specific *pf*LDH (black bars) and PDC5*corr* (grey bars) activities in IBB14LA1 measured in cultivations on xylose-(YX) and glucose-(YG) based media under aerobic (_AE) and anaerobic (_AN) conditions. Samples were taken after 24 or 72 h of cultivation time, as indicated. Data represent mean values from quadruplicate experiments, including biological and technical replicates. Error bars indicate standard deviations

Since limitations at the level of the *specific* rate of *pf*LDH seem unlikely, the observed change in lactic acid and ethanol formation when glucose or xylose was the substrate probably arose due to variation in the relative amount of LDH and PDC dependent on the sugar substrate. To investigate this, the *pf*LDH and the PDC5 activities were measured in the cell extract of IBB14LA1. The results are summarized in Fig. 4. Please note, because the *pf*LDH competed with the PDC-ADH for pyruvate and NADH under the PDC assay conditions, the PDC activities reported for IBB14LA1 are PDC*corr* values, which were derived as described in the “Methods” section. As shown in Fig. 4, the activities of *pf*LDH and PDC*corr* measured after 24 h of anaerobic fermentation were similar to each other and higher on glucose (~ 0.8 U mg_crude cell protein_^-1^) as compared to xylose (~ 0.3 U mg_crude cell protein_^-1^). Based on the higher kinetic efficiency of the *pf*LDH as compared to the PDC5, this means that after 24 h, the amount of PDC5 protein in the cell must have exceeded that of the *pf*LDH significantly. When measuring the PDC activities in the parent strain (IBB10B05, Additional file 2: Fig. S1), no influence of the carbon source on the PDC activity was detected. It is tempting to speculate, therefore, that in the case of IBB14LA1, the higher PDC*corr* activity and the presumed relative increase in the PDC5/*pf*LDH protein ratio in the cell when glucose was used instead of xylose were indeed responsible for the increase in *Y*_EtOH_ and the relative decrease in *Y*_LA_ with glucose as the substrate (Fig. 3a). However, the *pf*LDH activity was similarly increased as the PDC activity in the presence of glucose (Fig. 4). Thus, further evidence (e.g., from transcriptome analysis) must be provided, to overcome the problem of precise quantitation of *pf*LDH and PDC activities present next to each other in the cell extract of IBB14LA1, and elucidate in full the carbon source dependency of lactic acid production in xylose-fermenting *S. cerevisiae*.

Time courses of anaerobic xylose fermentations using IBB14LA1 show a decline in lactic acid productivity after approximately 50 h of cultivation time (Fig. 2a). This effect was shown to be more pronounced when fermentations were done without pH stabilization [20]. To elucidate the underlying reasons, enzyme activities were additionally determined after 72 h of xylose fermentation. As compared to the 24 h sample, the PDC*corr* and *pf*LDH activities were 3-fold increased and 2-fold decreased, respectively (Fig. 4). Explanation for the observed decline in lactic acid productivity is thus suggested (Fig. 2a and [20]). Earlier studies of native strains of *S. cerevisiae* strains showed that a lack in PDC1 protein in the cell triggers upregulation of the *pdc5* gene expression [26, 35]. Evidence from this study indicates that, under anaerobic conditions, the *pdc1*/*pdc5* regulation pattern is also triggered on xylose in recombinant xylose-fermenting *S. cerevisiae*. Results further suggest that *pdc1* gene expression (here resulting in *pf*LDH production) is shut down with the onset of the expression of the PDC5.

### The Q_LA_ in IBB14LA1 is dependent on the supply of oxygen: evidence that xylose does not suppress the respiratory response as glucose does

Figure 2 further shows the time courses of aerobic substrate conversions using IBB14LA1. The physiological parameters calculated from the data are summarized in Additional file 1: Table S1, and *Y*_LA_, *Y*_EtOH_, and Q_LA_ are compared in Fig. 3a. In aerobic glucose conversions, IBB14LA1 produced similar final lactic acid titers (~ 10 g L^−1^, Fig. 2b) and *Y*_LA_ (0.19 g g_Glc_^−1^) as in anaerobic fermentations. The ethanol production was decreased (~ 11 g L^−1^ *Y*_EtOH_ and 0.23 g g_Glc_^−1^). The parameter most influenced by the addition of oxygen was Q_LA_, which increased from 0.38 to 0.81 g L^−1^ h^−1^ (Fig. 3a). When oxygen was supplied to xylose conversions, a severe decrease in lactic acid formation was observed. Ethanol production ceased completely (Figs. 2a, 3a). Considering the biomass yields in experiments without addition of CaCO_3_ (*Y*_Biomass_ ~ 0.24 g g_Xyl_^−1^, [20]) and the cell dry weight concentration after 24 h of cultivation (c_BM_ 9.5 g L^−1^ in aerobic and 3.3 g L^−1^ in anaerobic xylose conversions, Additional file 1: Table S1), it seems likely that carbon fluxes were mainly channeled towards biomass formation.

Consistent with the results of time course analysis, change from anerobic to aerobic glucose conversions did not affect the *pf*LDH activity, but resulted in a significant (5-fold) decrease in PDC*corr* activity (Fig. 4). In aerobic xylose conversions, no PDC*corr* activity was detectable, explaining the absence of ethanol formation (Figs. 2a, 3a). Interestingly, however, *pf*LDH activity was increased 2.3-fold as compared to anaerobic fermentations (Fig. 4), despite the observed drop in lactic acid production (Figs. 2a, 3a). This indicates that, at the pyruvate branching point, a factor additional to the PDC and *pf*LDH influences the distribution of metabolic fluxes when oxygen was provided. Data derived from chemostat cultures, as well as evidence from proteome, transcriptome, and flux balance analyses, suggest that xylose-to-ethanol converting strains of *S. cerevisiae* show Crabtree-negative characteristics when cultivated on xylose under aerobic conditions [37–40]. This implies that even at high xylose concentrations, the expression of the TCA cycle enzymes and of the respiratory enzymes is not repressed, as it is observed for glucose (the so-called Crabtree effect) [37, 41]. In aerobic xylose conversions using IBB14LA1, the pyruvate is therefore likely channeled away from the *pf*LDH-catalyzed reaction towards the TCA cycle, resulting in low lactic acid production, despite the presence of *pf*LDH activity in the cell (Figs. 2a, 4). That lactic acid production was measurable at all in IBB14LA1, and also in IBB14LA1_5 (Fig. 3a, b) was probably a consequence of the evolution background of these two strains [28]. The parent strain of IBB14LA1 and IBB14LA1_5, strain IBB10B05, was evolutionary engineered with the aim of accelerating xylose-to-ethanol conversion and facilitating anaerobic growth on xylose [28]. This resulted in a strongly enhanced recognition of xylose as fermentable sugar [28].

An interesting finding, revealed through the enzyme activity measurements in Fig. 4, was that switching from anaerobic and aerobic conditions resulted in a strong decrease (glucose) or even a disappearance (xylose) of PDC*corr* activity. The parent strain IBB10B05 with intact *pdc1* gene also showed a decrease in PDC activity in aerobic as compared to anaerobic conversions, but to a much lower extent (Additional file 2: Fig. S1). This, together with the decreased (glucose) or lack of (xylose) ethanol production in aerobic cultivations of IBB14LA1, provides good evidence that, when oxygen is present, the lack of PDC1 protein in strain IBB14LA1 does not trigger the *pdc5* gene expression to increase, as it was observed in anaerobic fermentations of IBB14LA1 in here (Figs. 2a, b, 4) and with other *S. cerevisiae* strains in literature [26, 35]. Although its underlying reason is not yet fully understood, the effect carries implications for further strain engineering, wherein a high *pf*LDH activity at basal amounts of PDC activity seems to be desirable, as will be described hereinafter.

### Xylose-to-lactic acid conversion by strain IBB14LA1_5 harboring a disrupted pyruvate-to-ethanol pathway: evidence for a role of oxygen in optimizing *Y*_LA_ and Q_LA_

**Fig. 5:**
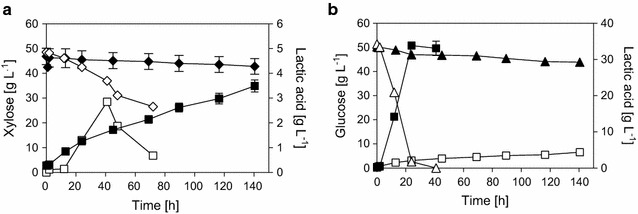
Time courses of xylose (**a**) and glucose (**b**) conversions using IBB14LA1_5. Depicted are the xylose (**a**, diamonds) and glucose (**b**, triangles) consumption and the lactic acid (**a**, **b**, squares) production in anaerobic (**a**, **b**, filled symbols) and aerobic (**a**, **b**, empty symbols) conversion experiments. Data and error bars represent mean values and the spread of biological duplicates. Data for from anaerobic conversions and aerobic glucose conversions are taken from [20]

The strain IBB14LA1_5 was also characterized in anaerobic and aerobic conversions of glucose and xylose. The results are depicted in Fig. 5. The physiological parameters of xylose and glucose conversions are summarized in Table 1 and Additional file 3: Table S2, respectively. The *Y*_LA_, *Y*_EtOH_ and Q_LA_ are compared in Fig. 3b, which showed that knockout of the *pdc5* efficiently diminished ethanol formation (*Y*_EtOH_ ≤ 0.04 g g^−1^).

**Table 1:** Physiological parameters of strain IBB14LA1_5 in anaerobic, aerobic and microaerophilic xylose-to-lactic acid conversions

Conditions	YX_AN	YX_AE^a^	YX_MA	
c_Xyl_/c_LA_ [g L^−1^]^b^	4.0/3.5	22.0 (11.6)/0.7 (2.9)	15.5/7.8	

Conditions	YX_AN	YX_AE^a^	YX_MA	
			Phase I^e^ (0–27 h)	Phase II (27–143 h)
Q_Xyl_/Q_LA_ [g L^−1^ h^−1^]	0.03 ± 0.01/0.03 ± 0.00	0.25 ± 0.01/0.05 ± 0.00	0.31 ± 0.07/0.11 ± 0.00	0.12 ± 0.00/0.04 ± 0.01
*Y*_LA_ [g g_Xyl_^−1^]	0.80 ± 0.05	0.03 ± 0.01 (0.25 ± 0.01)	0.24 ± 0.04	0.38 ± 0.01
*Y*_Ethanol_ [g g_Xyl_^−1^]	0.04 ± 0.01	*n.d.*	*n.d.*	*n.d.*
*Y*_Glycerol_ [g g_Xyl_^−1^]	*n.d.*	*n.d.*	*n.d.*	*n.d.*
*Y*_Xylitol_ [g g_Xyl_^−1^]	0.08 ± 0.01	0.1 ± 0.00 (0.1 ± 0.00)	*n.d.*	0.02 ± 0.00
*Y*_Acetate_ [g g_Xyl_^−1^]	0.04 ± 0.01	*n.d.*	0.18 ± 0.04	0.10 ± 0.01
*Y*_Biomass_ [g g_Xyl_^−1^]	*n.a.* ^c^	*n.a.* ^c^	0.31 ± 0.03	*n.d.*
*Y*_Pyruvate_ [g g_Xyl_^−1^]	*n.d.*	0.02 ± 0.00	*n.d.*	*n.d.*
*Y*_CO2_ [g g_Xyl_^−1^]	*n.a.*	*n.a.*	0.25 ± 0.02	0.58 ± 0.01
C-recovery [%]	100.8 ± 7.0	*n.a.* ^d^	94.6 ± 2.2	99.6 ± 4.9

*n.d.* not detectable, *n.a.* not analyzed

^a^Parameters were determined for the entire fermentation time (72 h). Parameters in brackets were determined for 41 h of fermentation, when lactic acid concentration was the highest (see time courses in Fig. 5a). Q_LA_ and Q_Glc_ represent initial volumetric consumption and production rates and are therefore unaffected by the lactic acid uptake

^b^Consumed xylose and final lactic acid titers

^c^*Y*_Biomass_ could not be determined due to addition of CaCO_3_ for pH stabilization

^d^Because of lactic acid uptake (Fig. 5a) C-recovery could not be determined

^e^Yields and mass balance were calculated on consumed xylose and ethanol

In anaerobic fermentations using IBB14LA1_5 (0.80 g g_Xyl_^−1^; 0.67 g g_Glc_^−1^) the effect of the carbon source on *Y*_LA_ was less pronounced as observed for IBB14LA1. Glycerol accumulation accounted for most of the carbon loss in glucose fermentations ([20] and Additional file 3: Table S2).

Addition of oxygen to glucose conversions resulted in a substantial boost in the conversion rates (Q_LA_ from 0.05 to 1.77 g g^−1^ L^−1^) without loss in *Y*_LA_ (0.69 g g_Glc_^−1^; Fig. 3b). In aerobic xylose conversions, the cells exhibited a lag phase (0–15 h of cultivation time), after which lactic acid was produced continuously until a peak concentration was reached (41 h of cultivation time, Fig. 5a). After this, lactic acid concentration decreased again (Fig. 5a). Under aerobic conditions, *S. cerevisiae* can consume and metabolize lactic acid [42]. At peak lactic acid concentration (41 h, Fig. 5a), Q_LA_ and *Y*_LA_ were 0.05 g g^−1^ h^−1^ and 0.25 g g_Xyl_^−1^, respectively. Thus, similar to conversions using the strain IBB14LA1, the addition of oxygen increased the glucose-to-lactic acid conversion by strain IBB14LA1_5 but decreased the xylose conversion in yields and productivities.

**Fig. 6:**
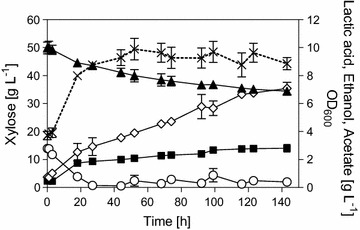
Time course of microaerophilic bioreactor conversions using strain IBB14LA1_5. Depicted is the conversion phase of xylose with a pO_2_ of ~ 2%. Data and error bars represent the mean values and the spread of biological duplicates. Symbols: Xylose—filled triangles; lactic acid—empty diamonds; ethanol—empty circles; acetate—filled squares; OD_600_—dashed lines and crosses

Evidence from anaerobic and aerobic glucose conversions (Fig. 3) therefore suggest that oxygen is necessary for achieving a high Q_LA_. The reason for this is likely the increased ATP generation through the electron transport chain, that enables the cells to meet the ATP requirements for maintenance, a task that *pdc*-negative lactic acid producing *S. cerevisiae* strains cannot fulfill by fermentation only [25, 27]. On xylose, however, aerobic conversions suffer from an insufficient repression of the respiratory response, resulting in a strong metabolic “pull” towards the TCA cycle and effectively channeling the pyruvate away from the *pf*LDH-catalyzed reaction. A similar effect has been described for IBB14LA1 above (Figs. 2, 4). Another drawback is the consumption of lactic acid by the cells (Fig. 5a). To increase cell viability by supplying oxygen, but avoid the above-mentioned disadvantages of fully aerobic conditions, the process set up was changed. Online regulation of the bioreactors facilitated adjustment of low oxygen levels (pO_2_ 2%) without changing the experimental setup in terms of the starting OD_600_. The time courses of microaerophilic conversions are depicted in Fig. 6. A summary of the physiological parameters is given in Table 1. Please note, because of the combined knock-out of the *pdc1* and *pdc5* gene, the strain IBB14LA1_5 does not grow on glucose [24, 26]. Cell propagation, accomplished in the bioreactor with 1 L working volume as described in the “Methods” section, was therefore performed on ethanol-based media. The time course in Fig. 6 shows only the conversion phase. The reaction proceeded in 2 phases: a transition phase (“phase I”; 0–27 h) where a residual amount of ethanol (2.8 g L^−1^) was still present and a second phase (“phase II”; 27–140 h) in which xylose was the only remaining carbon source (Table 1, Fig. 6). In phase I, Q_LA_ was fastest (Q_LA_ ~ 0.11 g L^−1^ h^−1^) and pronounced cell growth was observed (*Y*_Biomass_ 0.31 g g_Xyl_^−1^). *Y*_LA_ based on xylose and ethanol was 0.24 g g_Xyl+EtOH_^−1^. The *Y*_LA_ based on consumed xylose was only ~ 0.33 g g_Xyl_^−1^. One of the primarily formed by-products was acetate (*Y*_Acetate_ 0.18 g g_Xyl_^−1^), which is an intermediate of ethanol catabolism. In phase II (27–140 h), the conversion slowed down (Q_Xylose_ ~ 0.04 g L^−1^ h^−1^) and the *Y*_LA_ was ~ 0.38 g g_Xyl_^−1^.

Thus, microaerophilic conditions increased Q_LA_ (phase I) 2.2- and 3.7-fold as compared to aerobic and anaerobic conditions, respectively (Table 1). The *Y*_LA_ on xylose was 1.5-fold higher than the *Y*_LA_ in aerobic conversions at peak concentration (Fig. 5a), and it was half the *Y*_LA_ obtained in anaerobic fermentations (Table 1, Fig. 3b). Moreover, under microaerophilic conditions continued lactic acid formation was facilitated, effectively preventing lactic acid metabolization (Fig. 6).

Results from aerobic, anaerobic, and microaerophilic xylose conversions, that is, the increasing *Y*_LA_ with decreasing pO_2_ and the significantly enhanced Q_LA_ at 2% pO_2_ (Table 1), are in line with the recent findings from Jouhten et al. [41]. The authors showed that the switch from fully respiratory to respiratory-fermentative metabolism in wildtype yeast happens between 2.8 and 1% oxygen in the inlet gas. The authors analyzed the net distribution of carbon fluxes at the pyruvate branching point, and found that it shifted from ~ 60% through the pyruvate dehydrogenase (PDH, Fig. 1) at 2.8% oxygen to ~ 30% at 1% oxygen in the inlet gas [41]. The fluxes through the PDC showed exactly opposite behavior, going from 30% (1% oxygen) to 60% (2.8% oxygen) [41]. The study was conducted in a glucose-limited chemostat to prevent respiro-fermentative metabolism based on glucose repression, which makes it more comparable to xylose fermentation, where the recombinant yeast strains show Crabtree-negative characteristics [35–38]. These findings from literature support the hypothesis that the underlying reason for the increase in *Y*_LA_ with decreasing pO_2_, consistently observed for IBB14LA1 and IBB14LA1_5, is the repression of the fluxes through the respiratory pathways, which increases the availability of pyruvate for the *pf*LDH-catalyzed reaction. They further suggests that the high Q_LA_ in combination with the useful *Y*_LA_ observed in microaerophilic conversions by strain IBB14LA1_5 (Table 1) was caused by a beneficial distribution of metabolic fluxes, facilitating both sufficient ATP generation through the respirative metabolism and high lactic acid production through fermentative metabolism.

### Engineering targets for optimzation of xylose-to-lactic acid converting yeast strains

Aggregate results from the current study show that, in aerobic xylose conversions, the pyruvate-to-lactic acid pathway in strains IBB14LA1 and IBB14LA1_5 suffers from a low availability of pyruvate, caused by a strong metabolic pull through the respiratory pathways. Results from microaerophilic conversion studies further suggest that a combination of high Q_LA_ and *Y*_LA_ can be achieved by optimizing the distribution of the carbon fluxes between the PDH (~ 30%) and the PDC (~ 60%, [41]). Indeed downregulation, but not elimination, of the PDH activity has been shown to increase isobutanol production in a recombinant *S. cerevisiae* strain due to an increased pyruvate availability for the valine biosynthetic pathway enzymes [43]. Another approach for pyruvate channeling has been suggested by Kim et al. [44]. The authors linked a LDH enzyme directly to the pyruvate kinase by cohesin–dockerin interaction, to capture the pyruvate directly in the cytosol before it can enter the respiratory pathways in the mitochondria [44].

Results from enzyme activity measurements in IBB14LA1 further suggest that the *pf*LDH/PDC5 ratio is critical to shift the distribution of the carbon fluxes from the ethanol to the lactic acid producing pathway. Because *pdc1* and *pdc5* knockout mutants exhibit reduced biomass growth and cell viability [20, 24–26], optimization of the gene expression level of the *pfldh* and *pdc5* might be a more successful approach. Thus, it might be possible to combine high cell viability (by allowing basal PDC5 activity for cytosolic acetyl-coA production [24]) with good lactic acid yields and productivities (by shifting the distribution of the carbon fluxes towards the *pf*LDH-catalyzed reaction).

## Concluding remarks

This study shows that the distribution of carbon fluxes at the pyruvate branching point in *S. cerevisiae* with and without intact ethanol metabolism is dependent on carbon source and oxygen. Evidence from time course analyses and enzyme activity measurements of IBB14LA1 and IBB14LA1_5 suggest that the underlying reason for this is the lack of suppression of respiratory response on xylose in combination with differential induction of gene expression, most importantly the PDC5, the PDH and the *pf*LDH genes. This places new targets for strain engineering to increase yields and productivities and thus, advance renewable lactic acid production at scale.

## Additional files

**Additional file 1: Table S1.** Physiological parameters of strain IBB14LA1 in glucose and xylose conversions under anaerobic and aerobic conditions.

**Additional file 2: Fig. S1.** The PDC activity of IBB10B05 measured in cultivations on xylose- (YX) and glucose-(YG) based media under aerobic (_AE) and anaerobic (_AN) conditions. Samples were taken after 24 or 27 h of cultivation time, as indicated. Data represent mean values from quadruplicate experiments, including biological and technical replicates. Error bars indicated standard deviations.

**Additional file 3: Table S2.** Physiological parameters of strain IBB14LA1_5 in glucose conversions under anaerobic and aerobic conditions.
