# Supplementary material for: l-Lactic acid production from glucose and xylose with engineered strains of Saccharomyces cerevisiae: aeration and carbon source influence yields and productivities
Source: Microb Cell Fact. 2018 Apr 11;17:59. doi: 10.1186/s12934-018-0905-z (PMC5894196; doi:10.1186/s12934-018-0905-z)
Supplement: Supplementary file 1 — Additional file 1: Table S1. Physiological parameters of strain IBB14LA1 in glucose and xylose conversions under anaerobic and aerobic conditions. [file 12934_2018_905_MOESM1_ESM.docx]

**Additional file 1: Table S1:** Physiological parameters of strain IBB14LA1 in glucose and xylose conversions under anaerobic and aerobic conditions.

|  |  | YG_AN^a1)^ |  | YX_AN^a2)^ |  | YG_AE^a3)^ |  | YX_AE^a4)^ |
| --- | --- | --- | --- | --- | --- | --- | --- | --- |
| c_Glc/Xyl_ / c_LA_  [g L^-1^]^b)^ |  | 54.6 /  10.8 |  | 22.0 /  6.1 |  | 50.7 /  10.0 |  | 18.4 /  1.5 |
| c_BM_ [g L^-1^]^c)^ |  | 5.2 |  | 3.3 |  | 13.8 |  | 9.5 |
| Q_Glc/Xyl_ / Q_LA_  [g L^-1^ h^-1^] |  | 2.06 ± 0.01 /  0.38 ± 0.01 |  | 0.20 ± 0.00 /  0.06 ± 0.01 |  | 3.30 ± 0.14 /  0.81 ± 0.18 |  | 0.22 ± 0.01 /  0.01 ± 0.00 |
| *Y*_LA_ [g g_Glc/Xyl_^-1^] |  | 0.18 ± 0.00 |  | 0.27 ± 0.02 |  | 0.19 ± 0.03 |  | 0.08 ± 0.01 |
| *Y*_Ethanol_ [g g_Glc/Xyl_^-1^] |  | 0.32 ± 0.01 |  | 0.15 ± 0.02 |  | 0.23 ± 0.00 |  | *n.d.* |
| *Y*_Glycerol_ [g g_Glc/Xyl_^-1^] |  | 0.06 ± 0.0 |  | 0.02 ± 0.00 |  | 0.11 ± 0.00 |  | *n.d.* |
| *Y*_Xylitol_ [g g_Glc/Xyl_^-1^] |  | *n.d.* |  | 0.19 ± 0.01 |  | *n.d.* |  | *n.d.* |
| *Y*_Acetate_ [g g_Glc/Xyl_^-1^] |  | *n.d.* |  | 0.03 ± 0.01 |  | 0.10 ± 0.01 |  | *n.d.* |
| *Y*_Pyruvate_ [g g_Glc/Xyl_^-1^] |  | *n.d.* |  | *n.d.* |  | *n.d.* |  | *n.d.* |
| C-recovery^d)^ [%] |  | 86.1 ± 1.9 |  | 80.7 ± 5.3 |  | 89.2 ± 2.1 ^c)^ |  | *n.d.* |

*n.d. – not detectable*

a) Parameters were determined for 52 h (a1), 142 h (a2), 24 h (a3) and 100 h (a4) of fermentation

b) Consumed glucose or xylose and final LA titer.

c) Cell dry weight determined after 24 h of cultivation. Data were generated during enzyme activity measurements, as described in the methods section. A cell dry weight to OD_600_ ratio of 0.52 was applied.

d) C-recovery exclude biomass yields, which could not be determined due to addition of CaCO_3_.
