# Supplementary figures and images for: l-Lactic acid production from glucose and xylose with engineered strains of Saccharomyces cerevisiae: aeration and carbon source influence yields and productivities
Source: Microb Cell Fact. 2018 Apr 11;17:59. doi: 10.1186/s12934-018-0905-z (PMC5894196; doi:10.1186/s12934-018-0905-z)

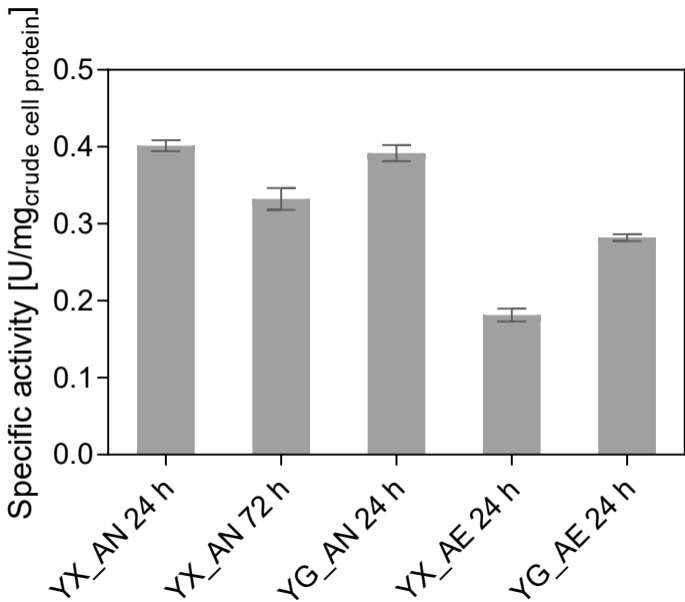

Supplement: Supplementary file 2 — Additional file 2: Fig. S1. The PDC activity of IBB10B05 measured in cultivations on xylose- (YX) and glucose-(YG) based media under aerobic (_AE) and anaerobic (_AN) conditions. Samples were taken after 24 or 27 h of cultivation time, as indicated. Data represent mean values from quadruplicate experiments, including biological and technical replicates. Error bars indicated standard deviations. [file 12934_2018_905_MOESM2_ESM.pdf]
