# Supplementary material for: l-Lactic acid production from glucose and xylose with engineered strains of Saccharomyces cerevisiae: aeration and carbon source influence yields and productivities
Source: Microb Cell Fact. 2018 Apr 11;17:59. doi: 10.1186/s12934-018-0905-z (PMC5894196; doi:10.1186/s12934-018-0905-z)
Supplement: Supplementary file 3 — Additional file 3: Table S2. Physiological parameters of strain IBB14LA1_5 in glucose conversions under anaerobic and aerobic conditions. [file 12934_2018_905_MOESM3_ESM.docx]

**Additional file 2: Table S2:** Physiological parameters of strain IBB14LA1_5 in glucose conversions under anaerobic and aerobic conditions.

|  |  | YG_AN^a1)^ |  | YG_AE^a2)^ |
| --- | --- | --- | --- | --- |
| c_Glc/Xyl_ / c_LA_  [g L^-1^]^b)^ |  | 6.4 /  4.4 |  | 51.4/  33.0 |
| Q_Glc/Xyl_ / Q_LA_  [g L^-1^ h^-1^] |  | 0.07 ± 0.01 /  0.05 ± 0.01 |  | 2.5 ± 0.05 /  1.77 ± 0.04 |
| *Y*_LA_ [g g_Glc_^-1^] |  | 0.67 ± 0.04 |  | 0.69 ± 0.01 |
| *Y*_Ethanol_ [g g_Glc_^-1^] |  | 0.04 ± 0.01 |  | *n.d.* |
| *Y*_Glycerol_ [g g_Glc_^-1^] |  | 0.10 ± 0.00 |  | 0.06 ± 0.00 |
| *Y*_Xylitol_ [g g_Glc_^-1^] |  | 0.02 ± 0.00 |  | *n.d.* |
| *Y*_Acetate_ [g g_Glc_^-1^] |  | 0.03± 0.00 |  | *n.d.* |
| *Y*_Pyruvate_ [g g_Glc_^-1^] |  | *n.d.* |  | 0.09 ± 0.00 |
| C-recovery^c)^ [%] |  | 90.3 ± 6.1 |  | 83.7 ± 0.8 |

*n.d. – not detectable*

a) Parameters were determined for 140 h (a1) and 24 h (a2) of fermentation

b) Consumed glucose or xylose and final LA titer

c) C-recovery exclude biomass yields, which could not be determined due to addition of CaCO_3_
